# Chemical Component Optimization Based on Thermodynamic Calculation of Fe-1.93Mn-0.07Ni-1.96Cr-0.35Mo Ultra-High Strength Steel

**DOI:** 10.3390/ma12010065

**Published:** 2018-12-25

**Authors:** Yongli Chen, Xuejiao Zhou, Jianguo Huang

**Affiliations:** 1 School of Metallurgical and Materials Engineering, Chongqing University of Science and Technology, Chongqing 401331, China; 2 School of Metallurgy and Environment, Central South University, Changsha 410083, China; 3 Manufacture Department of Benxi Steel Plate Co., LTD, Benxi 117000, China; jianguohuang1314@163.com; 4 State key laboratory of Rolling and Automation, Northeastern University, Shenyang 110819, China

**Keywords:** ultra-high strength steel, thermodynamic calculation, phase diagram, chemical component optimization, rolling process, reduction

## Abstract

Due to the complex composition and high proportion of alloys in traditional ultra-high-strength steel, the dilemma caused by ultra-high strength and low toughness in casting and forging forming processes requiring subsequent heat treatment can be mitigated with an efficient and economical rolling process. In this work, a thermodynamic model is proposed to calculate the change in the mechanical response due to the thermal contribution based on alloy phase diagrams. The influence of alloy content on precision laws was analyzed, and the chemical component was optimized. A verification simulation without real experiment was conducted to study the potential and limitations of the alloy content on mechanical properties. The results showed that the main equilibrium phases and the phases’ chemical compounds were clarified. The influences of Ni, Mo, Cr, and W on transformation laws were elucidated in detail, and the main optimized composition was determined to be 0.23C, 1.96Si, 1.93Mn, 0.07Ni, 1.96Cr, and 0.35Mo. At a cooling rate of 10 °C/s, the content of optimized alloying element fully performed its role in steel, verifying that the chemical component system was in the optimal range. The thermodynamic models and our conclusions have the potential to be generalized for many other materials and process configurations without requiring extensive material testing.

## 1. Introduction

The traditional ultra-high strength steel (UHSS) alloy composition system is complex and high in alloy content [1,2]. The system is usually used to precipitate alloy carbide particles on a fine high carbon flake martensitic matrix to enhance the dispersion precipitation strengthening effect and to achieve ultra-high mechanical strength. However, due to the large proportion of alloy content, the toughness is lowered and does not match the ultra-high strength, so subsequent heat treatment, such as quenching and tempering or quenching and partitioning, is required [3,4,5,6]. Therefore, the UHSS traditional process challenge caused by the high cost and high consumption of alloy need to be solved. The rolling process is efficient and reduces alloy, and could be an effective method to solve the current problem [7]. Moreover, In order to optimize and design the reduction chemical component system and apply to rolling process, thermodynamic model calculation and analysis are important research methods for minimizing chemical component design and prediction of mechanical properties of UHSS.

The crystal structure of the precipitation phase of alloying elements varies with different rolling temperatures, and can include the body-centered cubic phase (bcc), face-centered-cubic phase (fcc), alloy carbide (MC), alloy carbonitride (M(C,N)), and other forms. In order to calculate the mixed entropy of each chemical component phase, Hillert et al. [8] constructed a double sublattice model of (A, B)a(C, D)c type, where A and B are substitute elements or atoms, which together occupy a sublattice position, whereas C and D are gap chemical components or atoms occupy another sublattice position, and the letters a and c represent the number of positions in various sublattices. Based on Hiller’s research, Sundman et al. [9] extended the (A, B)a(C, D)c model into a multi-lattice model that can accommodate an infinite number of chemical components. The model expression is:

(1) $$G_{m}=\sum_{I0}P_{I0}(Y)G_{I0}^{0}+RT\sum_{S}a^{S}\sum_{i}y_{i}^{s}\ln y_{i}^{s}+\sum_{Z>0}\sum_{IZ}P_{IZ}(Y)L_{IZ}$$

where $G_{I0}^{0}$ is the free energy of the compound *I*, *a*^s^ is site fraction of chemical component *a* on sublattice s, *P_IZ_* is the product of the positional scores of each chemical component, *L*_IZ_ is the interaction parameter, *IZ* is the order of the array of chemical components, and *I* in $y_{i}^{s}$ is chemical component is the lattice location score.

The above thermodynamic model has received substantial attention. Many prior studies [10,11,12,13,14,15,16,17,18] proposed mathematical models for microstructure calculation, mechanical properties, and continuous cooling transformation based on parameters such as phase composition and compound. Moreover, Egea et al. [19,20] proposed thermodynamic models and described the change in the mechanical properties of wires. Allen, Liu, and Wang et al. [21,22,23] analyzed the crystal plasticity model, the plasticity model of DP600, and the prediction model of Inconel 625. However, most of the research in this field aimed at solving and verifying the accuracy of the model based on experimental results and correcting the correlation coefficient of the model.

The overall goal of this work was to optimize the chemical components in the rolling process. Unlike the aforementioned studies, the thermodynamic mathematical model here was used to investigate the influences of alloy content on the phase diagram and precision laws. The chemical component system was optimized by reducing Ni, reducing Mo, removing W, increasing Cr, and obtaining the same phase diagram compared with the origin content. Finally, using common process parameters of cooling rate at 10 °C/s after direct hot rolling, the result showed that the content of optimized alloying element fully performed its role in steel, verifying that the chemical component system was in the optimal range. This paper offers a novel research method for ultra-high strength steel and provides theoretical and practical guidance for rolling production.

## 2. Thermodynamic Calculation Model

### 2.1. Microstructure and Phase Calculation Model

Due to the different chemical components composed of different alloying elements and the different volume fractions of the chemical components, the performance values of the specific phases of the chemical components will also be different. The relationship between the chemical components and the properties can be expressed by Equation (2) [24]. According to the compositional phase properties and volume fraction of the material, the general properties of the material can be calculated by using the mixing in Equation (3) [9].

(2) $$P=\underset{i}{\sum }x_{i}p_{i}^{o}+\sum_{i}\sum_{j>i}x_{i}x_{j}\sum_{v}\Omega_{ij}^{v}{\left(x_{i}-x_{j}\right)}^{v}$$

(3) $$P_{t}=x_{\alpha }P_{\alpha }^{}+x_{\gamma }P_{{}_{\gamma }}^{}+x_{\mathrm{III}}P_{\mathrm{III}}^{}$$

where *P* is the strength value of the phase; $p_{i}^{o}$ is the strength value of the pure phase; $\Omega_{ij}^{v}$ is the coefficient of the *v* phase; *x_i_* and *x_j_* are the mole fractions of the *i* and *j* chemical components, respectively; the correlation of the mixed phase of the experimental material is obtained by *P_t_* performance; *x*_α_ and *x*_γ_ are the molar fractions of the *α* phase and *γ* phase, respectively; *P_α_* is the strength of the α phase; *P*_γ_ is strength of the γ phase; and *P*_III_ is the strength of phase *III*.

### 2.2. Yield Strength Calculation Model

Based on the Hall–Petch grain strengthening in Equation (4) [11,25,26] and the Ashby–Orowan second phase precipitation strengthening in Equation (5) [12,27], the grain size and corresponding precipitation enhancement can be calculated. Considering the influence of process parameters such as temperature, stress and strain rate on mechanical properties, the yield strength of processing enhancement is calculated using the Equation (6) [28,29,30].

Grain strengthening:

(4) $$\sigma_{y}=\sigma_{0}+kd^{-1/2}$$

Second phase enhancement:

(5) $$\sigma_{\mathrm{ppt}}=0.84M(\frac{1.2Gb}{2\pi L})\ln\frac{r}{b}$$

Processing enhancement:

(6) $$\sigma (y,\dot{\varepsilon })=\sigma (y,{\dot{\varepsilon }}_{0}){\left(\varepsilon /{\dot{\varepsilon }}_{0}\right)}^{m}$$

where *σ_y_* is the yield strength of the material, *σ*_0_ is the grain boundary stress of the corresponding pure metal, *k* is the material correlation coefficient, *d* is the grain size, *σ*_ppt_ is the second phase strength enhancement, *m* is the material correlation coefficient, *G* is the shear modulus of material, *b* is the Burgers vector, *L* is the distance of the precipitation phase, *r* is the size of precipitation phase, $\sigma (y,\dot{\varepsilon })$ is the strength of the material after processing, $\sigma (y,{\dot{\varepsilon }}_{0})$ is the strength of material at strain rate of ${\dot{\varepsilon }}_{0}$, and *m* is the temperature state parameter.

### 2.3. Tensile Strength Calculation Model

According to the microstructure and phase calculation Equation (3), the mechanical properties of each phase can be obtained. For the γ phase, α phase and alloy precipitation phase with the topological relationship, the tensile Equation (7) [9,31,32] is used to calculate the tensile strength according to the property and volume fraction of each phase.

(7) $$\sigma_{b}=x_{\alpha }\sigma_{\alpha }^{}+x_{\gamma }\sigma_{\gamma }+x_{\mathrm{III}}\sigma_{\mathrm{III}}^{}$$

where *σ*_b_ is the tensile strength of the mixed phase, *x*_α,_ *x*_γ_, *x*_III_ are the molar fractions of α, γ and precipitated phase, respectively, and *σ*_α_, *σ*_γ_, and σ_III_ are the strength of α, γ and precipitated phase, respectively.

### 2.4. Phase Volume and Performance Calculation Model

The phase volume fraction at a certain cooling rate can be obtained by calculating the phase composition under continuous cooling conditions according to Equation (8) [33]. According to the chemical component and its volume fraction, the performance of the corresponding phase is calculated using Equation (1). Finally, according to the phase performance and volume fraction of the material, the overall performance of the material is calculated by the mixing law.

(8) $$x=\frac{V}{V_{\mathrm{eq}}(T)}=1-\exp\left(-\frac{\pi }{3}N_{r}G_{r}^{3}t^{4}\right)$$

where *x* is the phase volume fraction, *T* is the temperature, *V* is the volume fraction at temperature *T*, *V*_eq_ is the volume under equilibrium transition, *N*_r_ is the nucleation rate, *G*_r_ is the grain growth rate, and *t* is the transition time.

## 3. Experimental Materials and Method

Based on a bainitic steel chemical component of 0.23C-1.96Si-1.93Mn-0.14Ni-1.84Cr-0.43Mo-0.01W-bal.Fe (S, P ≤ 0.009, Nb + V + Ti + Al + Cu + B ≤ 0.15. wt%), using Thermo-Calc (V5.0, Royal Swedish Institute of Technology, Stockholm, Sweden) and JMatPro (V6.0, Sente Software Ltd., Guildford, UK) calculation software, the chemical composition was optimized and the performance was predicted and analyzed.

### 3.1. Chemical Composition Design and Optimization

Using the above calculation model and thermodynamic calculation software of Thermo-Calc (TCFE6 database), under the premise of not changing the basic phase in the equilibrium phase diagram and the other alloy contents were unchanged, the gradient value of the target alloy content is changed to calculate equilibrium phase properties of each alloy content. Then the precipitation variation of carbides in steel were studied. Finally, the alloy composition was finally optimized. The detailed process was as follows: (1) Calculate the temperature-equilibrium phase diagram of the original steel and analyze the phase composition and precipitation. (2) Study effects of the content of Ni, Mo, W and Cr on precipitation of corresponding carbides while the other alloy contents remain unchanged. (3) Compare the original and optimized equilibrium phase diagram of experimental steel, and analyze the influences of the alloy content on the phases.

### 3.2. Mechanical Properties Analysis

According to the above chemical composition optimized, the carbide composition and alloying carbide morphology were analyzed by JMatPro, and the influence of the main alloy contents in experimental steel on mechanical properties under equilibrium state was analyzed. Setting the other alloy contents to fixed values (the average value of the impossible value range) and the target alloy content as a variable, the influences of the chemical composition on phase composition content and mechanical properties were analyzed at common cooling rate of 10 °C/s in the Thermo Mechanical Control Process (TMCP) process of medium plate production.

## 4. Results and Discuss

### 4.1. Phase and Chemical Composition Optimization

#### 4.1.1. Equilibrium Phase Diagram of Original Bainitic Steel

**Figure 1:**
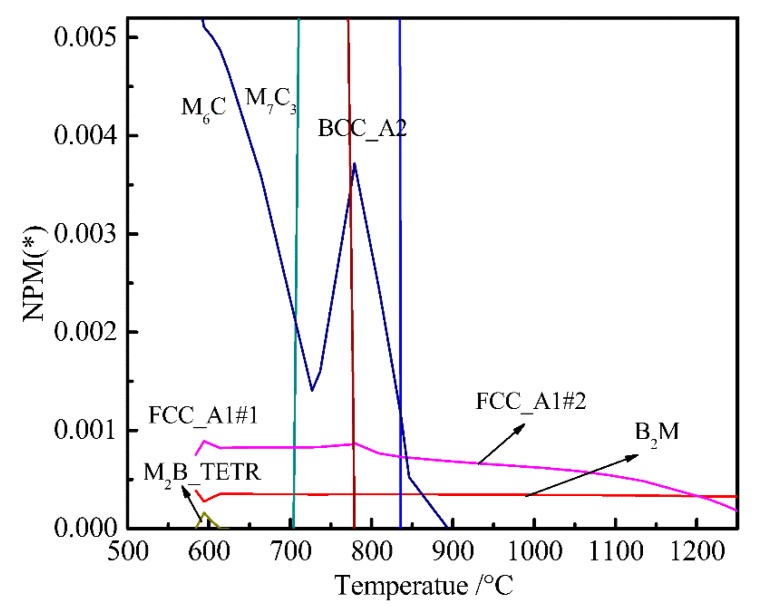
Temperature-chemical component equilibrium phase diagram of bainitic steel.

To determine the temperature-equilibrium phase of the original bainitic steel, we set the chemical composition of the experimental steel, set the temperature in the solid phase transition temperature between 20 and 1250 °C, and calculated the precipitation laws of alloy elements in the cooling process of γ→α. A partial enlargement of the temperature-number of moles of a phase per mole (T-NPM) is shown in Figure 1. Using the multi-lattice model, the crystal structure expression for each phase in the steel [34] was: (Fe, M_1_...M_i_, V, Nb)_a_ (C, N, Va_)c_. where M_1_...M_i_ is an alloying element in steel, and Va is a vacancy. *a* = 1 and *c* = 1 are fcc phases, and *a* = 1 and *c* = 3 are bcc phases. In the (Fe, M_1_...M_i_, V, Nb)a (C, N, Va)c lattice, the first sublattice is the regular lattice position and the second sublattice is the octahedral gap [9]. Alloying elements, such as Fe, M_i_, V, and Nb, can be substituted for each other in the sublattice, and alloying elements, such as C and N, can be substituted for each other in the vacancy gap. According to the (Fe, M_1_...M_i_, V, Nb)a(C, N, Va)c lattice model, B_2_M is mainly composed of Ti and B (Figure 1); FCC_A1#2 is mainly composed of Ti, C, Cr, and Nb compound (fcc crystal structure); and M_6_C is mainly a compound composed of Mo, Fe, Si, C, and Cr.

#### 4.1.2. Effect of Ni and Mo Contents on Equilibrium Phase

**Figure 2:**
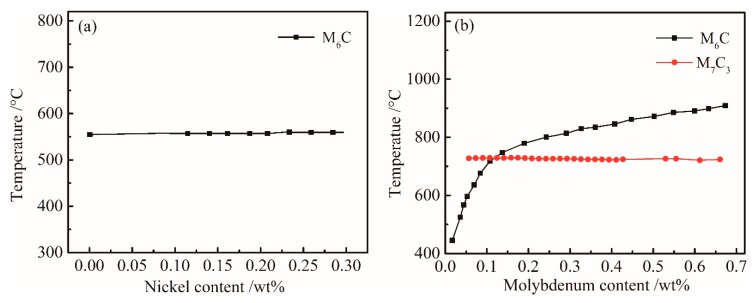
Influences of (**a**) Ni and (**b**) Mo content on the equilibrium phases of the bainitic steel.

In order to determine the influence of Ni content on the equilibrium phase, the variations in Ni and Mo contents were calculated to determine the variation in the composition-temperature equilibrium phase diagram of the experimental steel. As shown in Figure 2a, the effect of Ni content on M_6_C was minimal. When the Ni content changed from 0 to 0.3%, the solid solution temperature of M_6_C increased from 555 to 575 °C, but had little effect on. The alloying elements Mo and C have a strong affinity to form M_6_C and M_7_C_3_ alloys in steel. The effects of the change in Mo content on M_6_C and M_7_C_3_ and the solution temperature are shown in Figure 2b. With the increase in Mo content, the solid solution temperature of M_6_C type carbide increased, and the solution temperature of M_7_C_3_ type carbide increased. The dissolution of M_6_C type carbide at high temperatures caused the inhibition of γ grain growth to decrease. Comparing Figure 1 with Figure 2b, it can be seen that as the temperature decreased, a large amount of Mo-rich M_6_C_-_type carbide precipitated at temperatures below 900 °C. The precipitation of M_6_C type carbide during the controlled cooling process enhanced the precipitation strengthening effect. In the heating process, the appropriate amount of undissolved M_6_C will be able to prevent the growth of γ grains, thereby increasing the hardness and tensile strength of UHSS.

#### 4.1.3. Effect of Cr and W Content on Equilibrium Precipitation Phase

**Figure 3:**
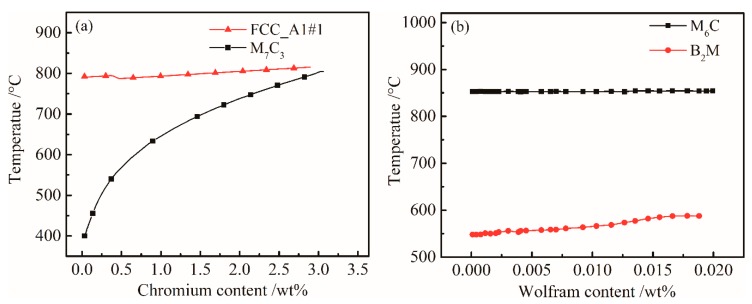
Influences of (**a**) Cr and (**b**) W content on the equilibrium phase diagram of the bainitic steel.

The effect of the content of Cr on M_7_C_3_ carbide in the steel is shown in Figure 3a. The solid solution temperature of FCC_A1#1 carbide increased with Cr content, and the M_7_C_3_ carbide with Cr is gradually dissolved into the matrix below 800 °C. In the controlled cooling process of the γ→α phase change, the M_7_C_3_ phase act absolutely as precipitation strengthening carbide, increasing the strength of UHSS.

In order to determine the influence of W content on the main equilibrium precipitate phases, the temperature-chemical component equilibrium phase diagram was analyzed. Figure 3b shows that the W content has slightly effect on M_6_C and B_2_M. Since the content of W was relatively low, and the mass fraction of M_6_C was small. From Figure 1, the B_2_M phase began to dissolve into the γ phase at temperatures above 1250 °C during the heating process. The mass of B_2_M gradually decreased with temperature during the cooling process. The overall content of B_2_M remained stable; significant changes only occurred around 580 °C. Figure 3b shows that B_2_M precipitates increased with W content at 500–600 °C.

**Figure 4:**
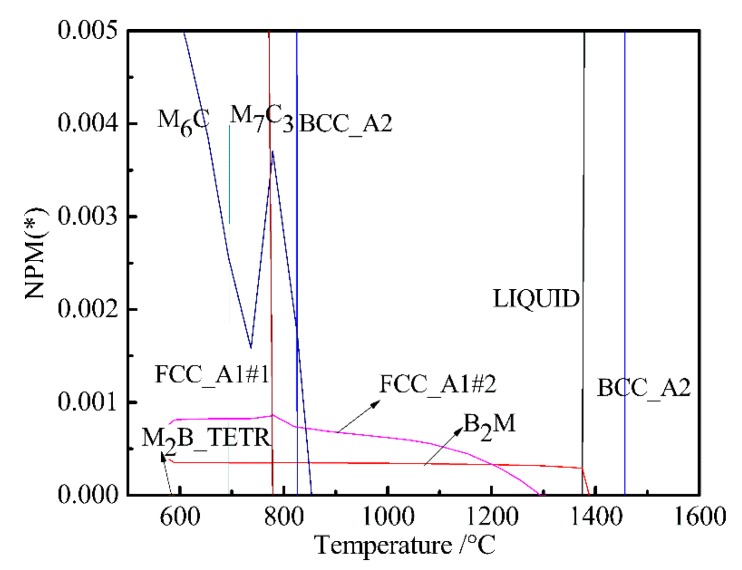
Partial magnification phase diagram of W-free bainite steel.

In order to more clearly analyze the effect of W on B_2_M, the temperature-chemical component equilibrium phase diagram of the W-free chemical component system was calculated and partially amplified as shown in Figure 4. Comparing Figure 1 with Figure 4, we can see the effect of W on FCC_A1#2, B_2_M, and M_2_B_TERT. It can be seen from Figure 1 that the mass of FCC_A1#2 and M2B_TETR alloy phases increased with decreasing B_2_M content at around 580 °C. The mass fraction of FCC_A1#2 and M2B_TETR decreased to a minimum with the increase in B_2_M. As can be seen from Figure 4, the content of FCC_A1#2 decreased and the content of B_2_M increased at about 580 °C; when the content of FCC_A1#2 decreased to a minimum, the content of B_2_M increased to the maximum.

**Figure 5:**
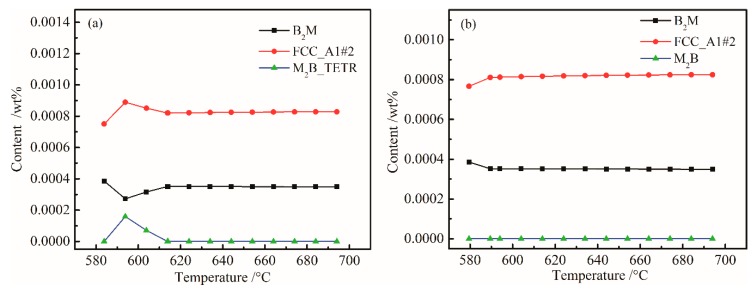
Plots of B_2_M, FCC_A1#2, and M_2_B_TETR content versus temperature: (**a**) 0.01 wt % W and (**b**) W-free.

Figure 5 shows the change laws of the mass percent of B_2_M, FCC_A1#2 and M_2_B_TETR in the original steel and W-free steel at 580–695 °C. Through comparative analysis, we found that W determines the changes in B_2_M, FCC_A1#2 and M_2_B_TETR between 580 and 614 °C. W mainly forms M_2_B_TETR boride in steel, but this boride is unstable, only appearing from 580 to 614 °C, and then transforming into other types of carbides. Through the above calculation, the Ni and W contents can be minimized. However, since there was 0.13% (wt %) of Cu in the steel, in order to prevent the adverse effects of Cu, the Ni content was adjusted to a mass percent of 0.07%.

#### 4.1.4. Effect of the Optimized Chemical Component System on Balanced Phase Diagram

**Figure 6:**
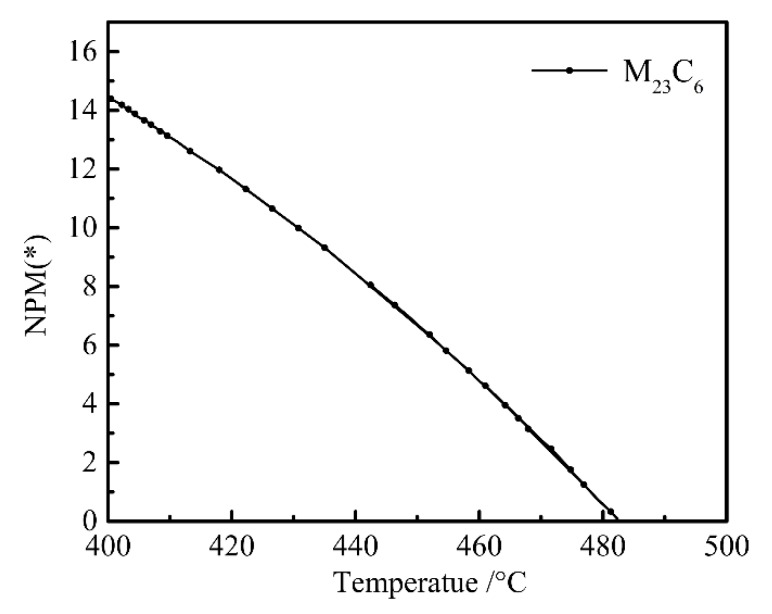
Plot of M_23_C_6_ content (wt%) versus temperature.

The reduction in Mo content promoted the formation of M_23_C_6_ phase in steel, while the increase in Cr content inhibited the production of M_23_C_6_ phase in steel [35]. After reducing Ni, Mo, and W, the chemical composition was adjusted to C 0.23, Si 1.96, Mn 1.93, Ni 0.07, Cr 1.84, Mo 0.35, Nb + V + Ti + Al + Cu + B ≤ 0.15, Fe bal. wt %), and the M_23_C_6_ precision is shown in Figure 6. When the Mo content was adjusted to 0.35%, M_23_C_6_ carbides appeared. M_23_C_6_ caused the hardenability of B to disappear [36], and the increase in Cr content produced the effect of avoiding M_23_C_6_. By calculation and comparison, we found that when the Cr content increased to 1.94%, the generation of M_23_C_6_ carbides was prevented.

**Figure 7:**
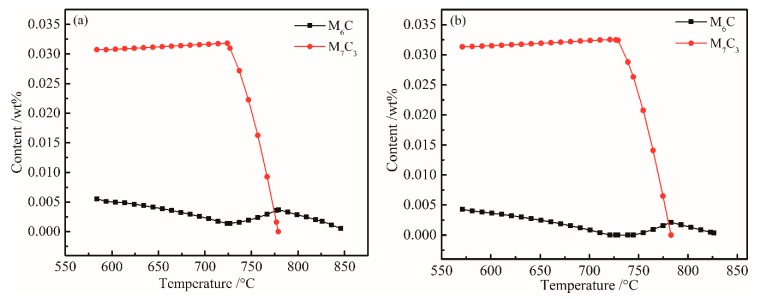
Plots of M_6_C and M_7_C_3_ content (wt%) of versus temperature. (**a**) Original; (**b**) Optimized.

The content of other alloys remained unchanged, and when the Cr content was further adjusted to 1.96 (wt %), a similar equilibrium phase diagram was obtained. To more accurately analyze the influence of Cr and Mo content changes on M_6_C and M_7_C_3_, the equilibrium phase diagrams before and after optimization are depicted in Figure 7. When the chemical composition was adjusted to C 0.23, Si 1.96, Mn 1.93, Ni 0.07, Cr 1.96, Mo 0.35, Nb + V + Ti + Al + Cu + B ≤ 0.15, Fe bal. wt %), an equilibrium phase diagram was obtained.

Comparing Figure 7a,b, the main phase composition and temperature precipitation characteristics did not change much, but the alloy content significantly decreased. The precision of the new chemical component system is consistent with the temperature window of the rolling process, and the new chemical component system meets the design goal of reducing the hot rolling process.

### 4.2. Effect of Alloy Content on Mechanical Properties

**Figure 8:**
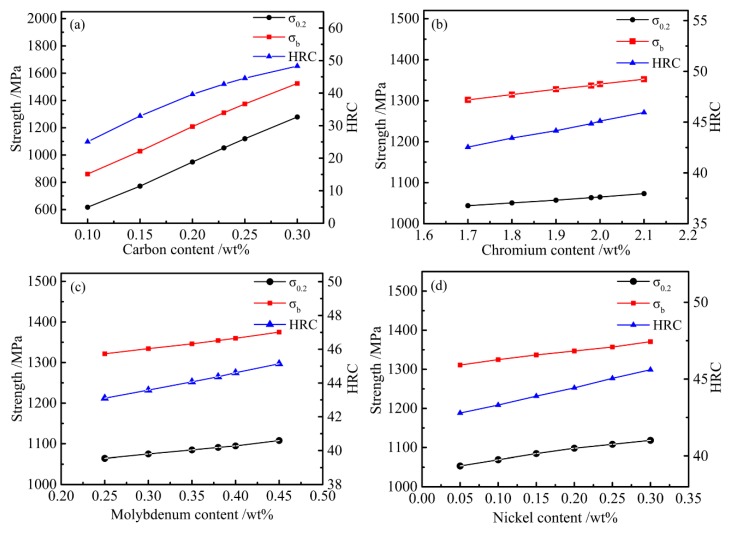
Influences of alloy content on the mechanical properties. (**a**) C; (**b**) Cr; (**c**) Mo; (**d**) Ni.

In order to control the smelting process and estimate TMCP parameters and mechanical properties, the calculation cooling rate was set to 10 °C/s because the common hot rolled products—hot rolled medium plate (*h* = 10–20 mm) and hot rolled bar (*D* = 15–25 mm)—are air cooled (CR) with a cooling rate of 5–15 °C/s after the direct hot rolling process. We varied the main alloying elements within the composition range (C 0.1–0.3, Cr 1.7–2.1, Mo 0.25–0.45, and Ni 0.05–0.3 wt %) to study the effect of alloy content on mechanical properties. The results can be drawn from Figure 8. With increasing carbon content, the change in ferrite and bainite was more obvious, as determined from Equations (4)–(6), As can be seen from Figure 8a, when the carbon content increased from 0.1% to 0.3%, the yield strength, tensile strength, and HRC increased from 617 to 1279 MPa, 860 to 1524 MPa, and 25 to 48, respectively. For each 0.05% increase in carbon, tensile strength, yield strength, and hardness linearly increased 168 MPa, 167 MPa, and 5.5, respectively. Comparing Figure 8b–d when the content of other alloying elements changed within the range of designed optimization values, the change in carbon content considerably influenced the strength performance. Because the change in carbon content directly affects the type of precipitates, as well as the amount of carbide precipitation of other alloys, which affects the precipitation strengthening, the strengthening effect on other alloys was ultimately affected. Considering strengthening effects on other alloys, a 0.23% carbon content was found to be the most appropriate.

According our analytical methods, the effects of the alloy strengthening on the mechanical properties due to Cr, Mo, and Ni were studied. It can be seen from Figure 8b–d that as the alloy content increased, their corresponding tensile strength, yield strength, and hardness increased, but the increase was small and insignificant. The analysis results show that the above optimized alloying elements fully strengthened the steel. If the alloy content continued to increase, its strengthening was not obvious. The chemical component system was in the optimal range (C 0.23, Si 1.96, Mn 1.93, Ni 0.07, Cr 1.96, Mo 0.35, Nb + V + Ti + Al + Cu + B ≤ 0.15, Fe bal.; wt %). The result proves that the above designed composition is feasible.

## 5. Conclusions

In order to optimize the reduction chemical component for UHSS during the rolling process, we used the thermodynamic mathematical model to study the effects of alloy, precipitation phase, and hot rolling temperature. The influence of the alloy content on precision laws was analyzed, and the chemical component system was optimized. The primary contributions and conclusions of this work are summarized as follows:

(1). The equilibrium phases in steel are B_2_M, BCC_A2, FCC_A1#1, FCCA1#2, M_6_C, M_7_C_3_, and M_2_B_TETR. B_2_M is a compound mainly composed of Ti and B. FCC_A1#2 is a compound mainly composed of Ti, C, Cr, and Nb. M_6_C is a compound mainly composed of Mo, Fe, Si, C, and Cr.
(2). When Ni content increased from 0 to 0.3%, M_6_C precision temperature increased from 555 and 575 °C, and the Ni content had little effect on FCC_A1#2 and M_6_C. Mo is a strong carbide element and forms M_6_C and M_7_C_3_ type carbides in UHSS. Mo content should not be too low, otherwise the strength of UHSS will decrease. M_6_C carbide with Cr increased with increasing Cr content. Below 800 °C, M_7_C_3_ carbide with Cr-rich gradually dissolved into the matrix. W mainly formed M_2_B_TETR borides. M_2_B_TETR can be converted with FCC_A1#2 and B_2_M in the temperature zone around 580 °C.
(3). The chemical component system was optimized by reducing Ni, reducing Mo, removing W, and increasing Cr, then we obtained the same phase diagram as obtained with the origin content. The optimized composition is C 0.23, Si 1.96, Mn 1.93, Ni 0.07, Cr 1.96, Mo 0.35, Nb + V + Ti + Al + Cu + B ≤ 0.15, Fe bal. (wt %). With a cooling rate of 10 °C/s, the optimized alloying system fully performed its strengthening role in the steel, and the chemical components were in the optimal range. The thermodynamic models and our conclusions have the potential to be generalized for many other materials and process configurations without requiring extensive material testing. However, a lack of a real experiment is unfortunate, and the limitations and practicality of this methodology will be verified in future experiments.
